# Absence of WASp Enhances Hematopoietic and Megakaryocytic Differentiation in a Human Embryonic Stem Cell Model

**DOI:** 10.1038/mt.2015.196

**Published:** 2015-12-08

**Authors:** Miguel G Toscano, Pilar Muñoz, Almudena Sánchez-Gilabert, Marién Cobo, Karim Benabdellah, Per Anderson, Verónica Ramos-Mejía, Pedro J Real, Olaf Neth, Agueda Molinos-Quintana, Philip D Gregory, Michael C Holmes, Francisco Martin

**Affiliations:** 1 Genomic Medicine Department, GENYO, Centre for Genomics and Oncological Research, Pfizer-University of Granada-Andalusian Regional Government, Parque Tecnológico Ciencias de la Salud, Granada, Spain; 2 Current address: Amarna Therapeutics S.L., Instituto Cartuja, C/ Leonardo da Vinci 19ª, Seville, Spain; 3 Current address: University College London-Institute of Child Health, London, UK; 4 Genomic Oncology Department, GENYO, Centre for Genomics and Oncological Research, Pfizer-University of Granada-Andalusian Regional Government, Parque Tecnológico Ciencias de la Salud, Granada, Spain; 5 Unidad de Enfermedades Infecciosas e Inmunopatologías Pediátricas, Hospitales Universitarios Virgen del Rocío, Instituto de Biomedicina de Sevilla, Sevilla, Spain; 6 UGC Hematología y Hemoterapia, Hospital Infantil Virgen del Rocío, Instituto de Biomedicina de Sevilla (IBIS)/CSIC/Universidad de Sevilla, Seville, Spain; 7 Sangamo BioSciences, Inc., Pt. Richmond Tech Center, Richmond, California, USA

## Abstract

The Wiskott-Aldrich syndrome (WAS) is an X-linked primary immunodeficiency caused by mutations in the *WAS* gene and characterized by severe thrombocytopenia. Although the role of WASp in terminally differentiated lymphocytes and myeloid cells is well characterized, its role in early hematopoietic differentiation and in platelets (Plts) biology is poorly understood. In the present manuscript, we have used zinc finger nucleases targeted to the *WAS* locus for the development of two isogenic *WAS* knockout (WASKO) human embryonic stem cell lines (hESCs). Upon hematopoietic differentiation, hESCs-WASKO generated increased ratios of CD34^+^CD45^+^ progenitors with altered responses to stem cell factor compared to hESCs-WT. When differentiated toward the megakaryocytic linage, hESCs-WASKO produced increased numbers of CD34^+^CD41^+^ progenitors, megakaryocytes (MKs), and Plts. hESCs-WASKO-derived MKs and Plts showed altered phenotype as well as defective responses to agonist, mimicking WAS patients MKs and Plts defects. Interestingly, the defects were more evident in WASp-deficient MKs than in WASp-deficient Plts. Importantly, ectopic *WAS* expression using lentiviral vectors restored normal Plts development and MKs responses. These data validate the AND-1_WASKO cell lines as a human cellular model for basic research and for preclinical studies for WAS.

## Introduction

Wiskott-Aldrich syndrome (WAS) is an X-linked primary immunodeficiency characterized by eczema, low Plts counts (microthrombocytopenia), recurrent infections, immunodeficiency, increased incidence of autoimmunity, and leukemia. The *WAS* gene is expressed exclusively in hematopoietic cells.^1,2,3^ Mutations that lead to the complete absence of WASp function cause the most severe phenotype with pronounced thrombocytopenia and abnormal lymphoid and myeloid function. However, the mutations that reduce the protein levels (or its function) result in milder variable phenotypes.^4,5^

The roles of *WAS* mutations in T cells, B cells, macrophages, dendritic cells, and natural killer cells have been extensively studied and are well characterized. Absence of WASp in these cell types alters actin polymerization causing problems on signaling, proliferation, migration, and phagocytosis. *WAS-*deficient mouse models^6,7,8^ and cell lines^9,10^ have been fundamental for the understanding of WASp function in all these cell types. However, *WAS* knockout (WASKO) mouse models do not mimic the Plts defects found in WAS patients making it difficult to study the function of WASp in MKs and Plts physiology.^11^ The role of WASp in Plt development remains largely unknown with contradictory results.^12,13^ In an elegant study, Sabri *et al*.^14^ identified a critical role of WASp in Plts biogenesis by inhibiting proplatelet formation through the Collagen I receptor. The same authors showed premature MK differentiation in the absence of WASp.^14^ Functional defects of WAS patient's Plts is also an open debate. Some authors described normal platelets activation, aggregation, α-granule secretion, and F-actin increase of WAS patient's Plts^15,16,17,18^ while other groups have reported mild or severe alteration of these functions.^19,20,21^ Similarly, very little is known about the role of WASp in early hematopoiesis with publications with apparent contradictory results.^6,22,23^

Human embryonic stem cells (hESCs) represent a unique tool to study early human development, for cell therapy and for developing *in vitro* models of human diseases. They can be differentiated into cell types from the three germ layers, providing an unlimited source of cells to study human development.^24,25^ Recently, the appearance of specific nucleases has made it possible to efficiently edit the genomes of most cell types.^26^ In particular, specific targeting of different loci using Zinc Finger Nucleases (ZFNs) have achieved efficient gene disruption, gene correction, and gene addition in several cell types including pluripotent stem cells.^27,28,29^ Gene edition of hESCs provides an excellent tool for modeling human monogenic diseases due to the possibility to obtain isogenic cell lines differing only in the desired mutations. These systems allow establishing a correlation genotype–phenotype without the interference of different genetic backgrounds as occurs when using induced pluripotent stem cells (iPSCs) derived directly from patients.

In the present manuscript, we have used ZFNs targeted to the *WAS* locus for the development of two WAS knockout (WASKO) hESC cell lines: AND-1_WASKO C1.1 and AND-1_WASKO C1.2. We have studied the role of WASp in early human hematopoiesis by comparing the *in vitro* generation of hematopoietic progenitors (CD34^+^CD45^+^) and mature CD45^+^ cells from AND-1_WASKO versus parental AND-1_WT. We have also investigated the role of WASp on megakaryopoiesis by analyzing MK progenitors, MKs, and Plts upon *in vitro* differentiation following a protocol described by Lu *et al*.^30^ This protocol allows the generation of functional Plts from hESCs. Finally, we have studied whether the phenotype observed in hESCs due to WAS mutation can be restored by ectopic expression of *WAS*.

## Results

### Generation of WASKO hESCs by homologous recombination using ZFNs

**Figure 1:**
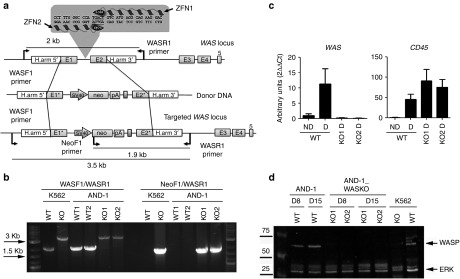
**Generation of hESCs-WASKO cell lines by targeting the *WAS* locus with ZFNs.** (**a**) Scheme depicting the strategy followed to mutate the *WAS* gene. Upper diagram illustrates the first five exons of the *WAS* locus (E1–E5), the sequences recognized by the ZFN pair (left ZFN and right ZFN, in the gray box), the homologous sequences used to promote homologous recombination (H.arm5' and H.arm3') and the primer pair used to identify the wild-type locus (rendering a 2-kb fragment). The middle diagram illustrates the donor DNA used to disrupt *WAS* expression. Exon 1 has a deletion in the ATG codon to block translation (E1*). Exon 2 includes mutations in the splicing acceptor site (E2*). A neo expression cassette (SV40-neo-pA) has been introduced to allow antibiotic selection. Lower diagram shows the ‘edited' *WAS* locus after homologous recombination with the donor DNA. Arrows indicate the primers used to identify those clones modified by homologous-directed recombination (HDR). (**b**) Successful gene editing of AND-1_WT cells. K562 cells wild-type (WT) and K562-WASKO (KO)^41^ have been used as negative and positive control of gene editing, respectively. Agarose gel shows the analysis to detect HDR in two G418-ressistant clones of the hESC line AND-1_WT. The band of 3.5 kb, present in both G418-resistant clones as well as in the K562 WASKO cells, indicates successful HDR. Also, the presence of the 1.9 Kb band in AND-1_WASKO as in the positive control indicates that the HDR happened in the right orientation. (**c**) *WAS* expression was abolished upon hematopoietic differentiation in AND-1_WASKO cells. *WAS* and *CD45* gene expression analysis performed by RT-qPCR of AND-1_WT (WT) and AND-1_WASKO clones (KO1 and KO2) before (ND) and after (D) 15 days of hematopoietic differentiation. *CD45* expression indicates the hematopoietic specification. As expected *WAS* expression was not detected in AND-1_WASKO clones. Values are mean of three experiments ± standard error of the mean. (**d**) Absence of WASp protein in hematopoietic differentiated AND-1_WASKO cells. Western blot analysis of protein extracts from AND-1_WT and AND-1_WASKO cells, obtained at days 8 (D8) and 15 (D15) of the hematopoietic differentiation. The membrane was hybridized with D1 (specific for WASp) and ERK (as loading control) antibodies. WASp protein was not detected in any of the AND-1_WASKO clones. K562 cells wild-type (WT) and K562-WASKO (KO) have been used as negative and positive control for WASp expression.

AND-1_WT1 cells^31^ were nucleofected with plasmids encoding ZFN targeted to the *WAS* intron1 (**Figure 1a**, top) and the donor DNA (**Figure 1a**, middle). Homologous-directed recombination (HDR)-driven modification of the *WAS* locus (**Figure 1a**, bottom) was evaluated by PCR. **Figure 1b** shows the PCR analysis of two clones; AND-1_WASKO_c1.1 (KO1) and AND-1_WASKO_c1.2 (KO2) (International Stem cell Registry, Spanish National Stem Cell Bank) using the primers depicted in **Figure 1a**. The gel shows the predicted bands if HDR occurred with the donor DNA (**Figure 1b**). AND-1_WASKO_c1.1 and AND-1_WASKO_c1.2 cells maintained similar phenotype (**Supplementary Figure S1**) and pluripotency (**Supplementary Figure S2**) as compared to the original AND-1_WT cells and to AND-1_WT2 cells (a clone obtained from AND-1_WT1, see Materials and Methods for details). Importantly, the karyotypes of both WASKO cell lines were identical to that of the original AND-1_WT1 cell line (**Supplementary Figure S3**).

The proper edition of the *WAS* locus should deplete *WAS* expression in AND-1_WASKO-derived hematopoietic cells. Indeed, while *WAS* expression could be clearly detected in AND-1_WT cells upon hematopoietic differentiation both at the mRNA (**Figure 1c** left) and protein levels (**Figure 1d**; AND-1, D8 and D15 WT lanes), AND-1_WASKO_c1.1 and AND-1_WASKO_c1.2 cells presented no detectable *WAS* mRNA (**Figure 1c**, left graph) or WASp protein (**Figure 1d**; AND-1_WASKO, D8 and D15; KO1 and KO2 lines). Of note, as expected, AND-1_WT cells did not express WASp in undifferentiated conditions (**Figure 1c**, left graph).

### WASKO hESCs generate increased number of hematopoietic progenitors and mature CD45^+^ cells

**Figure 2:**
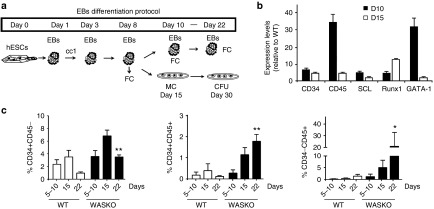
**Enhanced hematopoiesis of hESCs-WASKO cells**. (**a**) Scheme depicting the protocol for hematopoietic differentiation using embryoid bodies (EBs) formation. CC1: cytokine cocktail 1; (BMP-4 (25 ng/ml), Flt-3L (300 ng/ml), SCF (300 ng/ml), IL-3 (10 ng/ml), IL-6 (10 ng/ml), and G-CSF (50 ng/ml)). CFU, colony-forming units; FC, flow cytometry; MC, methylcellulose. (**b**) Graph showing AND-1_WASKO relative expression levels of hematopoietic markers (CD34 and CD45) and transcription factors SCL, RUNX1 and GATA-1 at day 10 (white bars) and 15 (black bars) of differentiation. Expression levels are shown relative to AND-1_WT (2^-ΔΔCt^ AND-1_WASKO/2^-ΔΔCt^ AND-1_WT). (**c**) Graph showing percentages of CD31^+^CD34^+^CD45^-^ (hemato-endothelial precursor cells, left graph), CD34^+^CD45^+^ (hematopoietic precursors; middle graph) and CD34^-^CD45^+^ (Mature hematopoietic cells; right graph) of AND-1_WT and AND-1_WASKO cells at different time points during the hematopoietic differentiation process. Data were obtained from cells gated based on size (FSC), granularity (SSC) and 7-AAD negative (viable) population as shown in **Supplementary Figure S12**. Data are mean ± standard error of the mean of at least four separate experiments using two AND-1_WT cell lines (AND-1_WT1 and AND-1_WT2) and two AND-1_WASKO (AND-1_WASKO_c1.1 and AND-1_WASKO c1.2). *P < 0.05; **P < 0.01.

We first studied the ability of *WAS*-deficient hESCs to generate hematopoietic cells. The different hESC lines were differentiated using the embryoid body (EB) system (**Figure 2a**) as described previously.^32^ mRNA was isolated at days 10 and 15 of differentiation and the expression levels of several hematopoietic-related genes analyzed by RT-qPCR (**Figure 2b**). Compared with AND-1_WT, AND-1_WASKO cells presented enhanced expression of the hematopoietic transcription factors SCL, RUNX1, and GATA-1 at day 10 and 15 of hematopoietic differentiation. We also observed an increase in the expression of CD34 and CD45 indicating a possible enhancement of the hematopoietic differentiation. To further investigate this finding, we measured the percentages of CD31^+^CD34^+^CD45^-^ (hemato-endothelial precursors), CD34^+^CD45^+^ (hematopoietic precursor cells), and mature CD34^-^CD45^+^ at different time points during the hematopoietic differentiation using the EB system (**Figure 2c**). Interestingly, AND-1_WASKO cells presented an increase in all these populations that reached significance at day 22. Similar results were obtained using the OP9 hematopoietic differentiation protocol (**Supplementary Figure S4**). These data indicate that the absence of WASp is altering hematopoietic differentiation generating higher numbers of progenitors and mature CD45^+^ cells. Although controversial, this data is in line with the observation that WASKO mice have higher numbers of progenitors and granulocytes in spleen and peripheral blood.^23^

### CD34^+^CD45^+^ cells derived from WASKO hESCs cells had altered responses to stem cell factor

**Figure 3:**
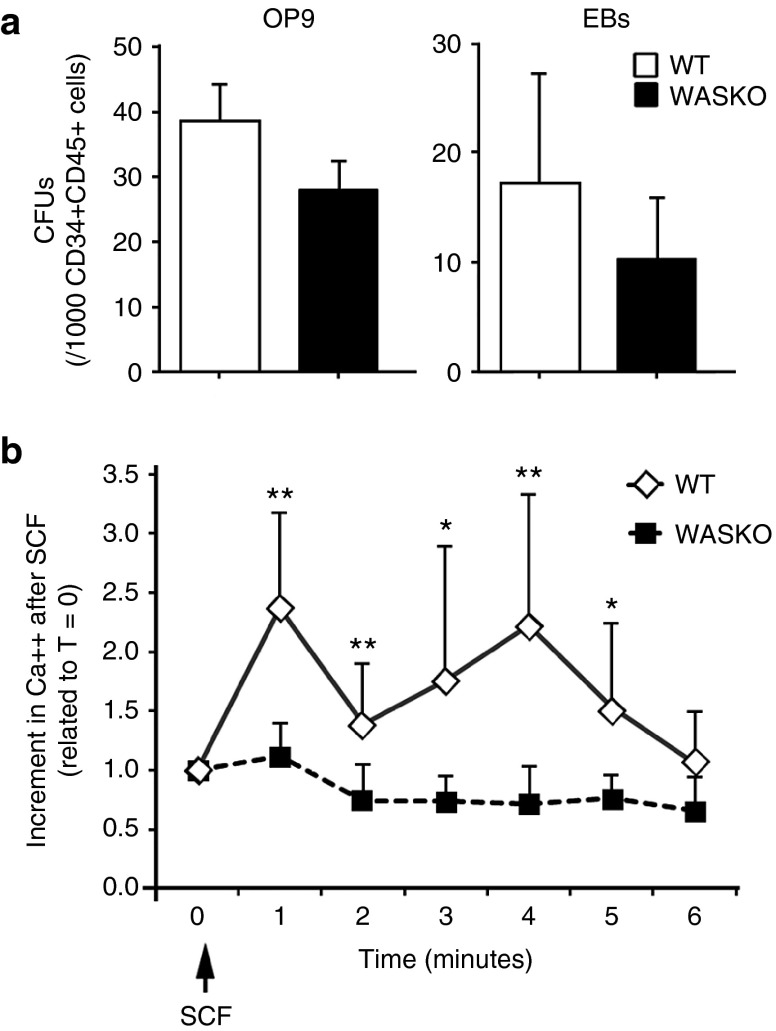
**Functional analysis of hESCs-WASKO derived CD34^+^CD45^+^ cells.** (**a**) Graphs showing colony-forming units (CFUs) potential of CD34^+^CD45^+^ derived from AND-1_WASKO-c1.1 (black bars) and AND-1_WT (white bars) on the OP9 differentiation system (left graph) or in the EBs system (right graphs). Day 15 (EBs) or day 10 (OP9) cells were harvested, analyzed for CD34 and CD45 expression and 1,000 CD45^+^CD34^+^ cells plated onto methylcellulose (see Materials and Methods for detail). (**b**) Graph representing intracellular Ca^++^ mobilization of CD34^+^CD45^+^ WASKO (squares) and WT (diamonds) cells in response to SCF. Day 15 cells (EBs) were harvested, washed, and prepared for Ca^++^ mobilization studies (see Materials and Methods for details). Cells were analyzed by flow cytometry with continuous acquisition for 30 seconds to measure background levels (*T* = 0). Acquisition was then paused to add the SCF (100 ng/ml; arrow) and immediately restart the continuous acquisition for 6 minutes. Ca^++^ flux is shown as the increment in MFI of SCF-stimulated cells at the different time points related to unstimulated cells (time = 0). Data are mean of at least three separate experiments ± standard error of the mean. *P < 0.05; **P < 0.01.

Mani *et al*.^22^ showed that WASp is involved in kit (stem cell factor (SCF)-receptor)-mediated signal transduction and required for kit-induced cellular Ca^++^ signaling and survival. We therefore studied whether WASKO CD34^+^CD45^+^ hematopoietic progenitor cells (HPCs) had altered responses to SCF (*i.e.*, reduced Ca^++^ flux) or reduced clonogenic potential in methylcellulose as previously showed by some authors.^33^ Although we found that HPCs derived from AND-1_WASKO cells (WASKO-HPCs) had a slight reduction in clonogenic potential (colony-forming units (CFU)/CD34^+^CD45^+^ cells) compared to the HPCs derived from AND-1_WT cells (WT-HPCs) both in the OP9 and EBs differentiation systems (**Figure 3a**), the data was not significant. These data is in agreement with Ingrungruanglert *et al*.^34^ showing a normal clonogenic potential of CD34+ cells derived from iPSCs from WAS patients. Interestingly, Ca^++^ Flux in response to SCF was significantly reduced in WASKO-HPCs, confirming the role of WASp in kit-mediated signals in hematopoietic progenitors (**Figure 3b**).

### WASKO hESCs cells differentiate into the megakaryocytic lineage generating WASKO-MKs and WASKO-Plts

**Figure 4:**
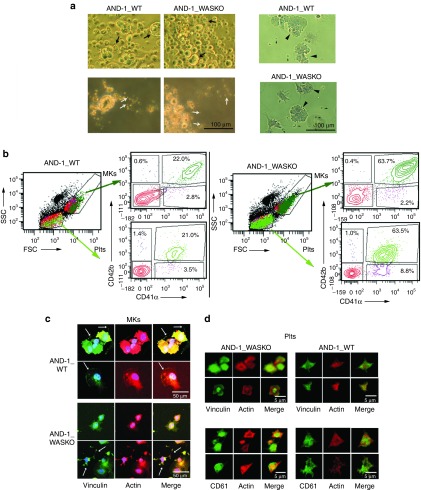
**Megakaryocytic differentiation and Plts production of hESCs-WASKO cells are not impaired**. (**a**) Morphological analysis of megakaryocytic differentiation cultures of AND-1_WT1 and AND-1_WASKO_c1.1 cells (see **Supplementary Figure S5** and Materials and Methods for details). Day 20 megakaryocytic cultures were photographed with a microscope Primo Vert (Carl Zeiss) on tissue culture plates (top-left panels), after collection (bottom-left panels) or after adhesion to fibrinogen coated slides and stained with Papanicolau (right panels). MKs and Plts-like structures are indicated by arrows. (**b**) Phenotypic characterization of cells contained in the megakaryocytic cultures. Cells were gated based on size (FSC) and granularity (SSC) into MK-region and Plts region (SSC/FSC plots). Each gate was further analyzed for expression of CD41 and CD42 (right plots at the side of the FSC/SSC plot). Large and granular, CD41^+^CD42^+^ cells were identified as MKs (SCC^high^FSC^high^CD41^+^CD42^+^; top plots, dark green). Small CD41^+^CD42^+^ cell fragments were identified as Plts (SSC^low^FSC^low^CD41^+^CD42^+^; bright green). (**c**) MK derived from AND-1_WT1 and AND-1_WASKO bind to fibrinogen and express CD61 and vinculin. Megakaryocytic cultures from AND-1_WT1 (top panels) and AND-1_WASKO_c1.1 (bottom panels) were collected on day 20, adhered to fibrinogen coated slides, activated with thrombin and immune-stained Phalloidin (red, actin), anti-vinculin (green) and 4',6-diamidino-2-phenylindole (DAPI) (blue). A Merge image is shown at the right of each set. (**d**) Platelets from AND-1_WT1 (top panels) and AND-1_WASKO_c1.1 (bottom panels) were collected on day 20 and treated as above. Samples were immune-stained with DAPI (Blue), Phalloidin (red) together with anti-vinculin (green) (top panels) or anti-CD61 (green) (bottom panels). A Merge image is shown at the right of each set. Confocal images were captured with a confocal microscope Zeiss LSM 880.

The low platelet counts (thrombocytopenia) found in WAS patients' blood can be restored to normal values after spleen removal^35^ suggesting that WAS patients do not have an intrinsic problems for Plt production. However, there are controversy in this matter.^14,33,34,36^ We used the AND-1_WASKO cells to study the role of WASp on megakaryocytic differentiation. The different hESCs lines were differentiated into the megakaryocytic lineage following the protocol pictured in **Supplementary Figure S5**.^30^ Lu *et al*. demonstrated that Plts derided from hESCs following this protocol responded to thrombin stimulation, formed microaggregates, and contribute to developing thrombi at sites of vascular injury in mice. Upon differentiation, human MK- and Plt-like structures were observed in the differentiation cultures of both cell lines both by microscopy (**Figure 4a**, arrows; **Supplementary Figure S6**) and flow cytometry (**Figure 4b**, arrows). The phenotypic characterization of the cells contained in our megakaryocytic cultures corroborated the presence of MKs and Plts (**Figure 4b**). Indeed, AND-1_WT and AND-1_WASKO cell lines generated large, granular, CD41^+^CD42^+^ MKs, (**Figure 4b**, top plots, dark green) and small Plts CD41^+^CD42^+^ (**Figure 4b**, bottom plots, bright green). Interestingly, AND-1_WASKO cell lines presented increased percentages of both MKs and Plts (**Figure 4b**, left versus right panels).

Additionally, when adhered to fibrinogen-coated slides and activated with thrombin, MKs from both WT and WASKO cell lines expressed CD61^+^ (**Supplementary Figure S7a**) and formed proplatelets and lamellipodia structures that were positive for actin and vinculin (**Figure 4c**, arrows and **Supplementary Figure S7b**). Interestingly, WASKO-MKs presented more irregular shapes and actin distribution than WT-MKs (**Figure 4c** and **Supplementary Figure S7b**). Plts derived from both cell lines were also CD61^+^ (**Figure 4d**, bottom panels and **Supplementary Figure S7c**), presented a trapezoidal structure as Plts from peripheral blood (**Supplementary Figure S8**) and had a clear colocalization of actin and vinculin (**Figure 4d**, top panels and **Supplementary Figure S7d**). As described for WAS patients, WASKO-Plts appeared slightly smaller and presented less protrusions compared with WT-Plts (**Figure 4d** and **Supplementary Figure S7c,d**). In summary, these data showed that AND-1_WASKO cells can efficiently generate MKs and produce Plts.

### Enhanced megakaryocytic differentiation of WASKO hESCs cells compared to WT hESCs

**Figure 5:**
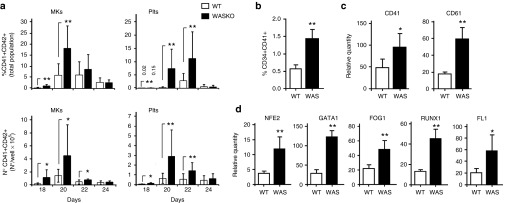
**Megakaryocytic specification and Plts production is enhanced in hESCs-WASKO cells.** (**a**) Enhanced MKs and Plts production in AND-1_WASKO compared to AND-1_WT cells. Graphs showing percentages (top) and total counts (bottom) of MKs (left) and Plts (right) derived from AND-1_WT (white bars) and AND-1_WASKO (black bars) cells at different days of megakaryocytic differentiation. Supernatant from OP9 cocultures were collected each 2 days, starting from day 18 and continued up to day 24. Cells were stained using CD41 and CD42 monoclonal antibodies and double positive cells considered as MKs and Plts as described in Figure 4. (**b**) Enhanced percentages of megakaryocytic progenitors in WASKO EBs. The EBs from AND-1_WT and AND-1_WASKO cells were dissociated on day 15, stained with anti-CD34 and anti-CD41 antibodies and analyzed by flow cytometry. Graph shows the percentage CD34^+^CD41^+^ cells identifying the MK progenitors. **P < 0.01. (**c**) and (**d**) Gene expression analysis of AND-1_WT (white bars) and AND-1_WASKO (Black bars) cells on EBs at day 15 of differentiation. Two MK specific surface markers (CD41 and CD61) (**c**) and five transcription factors associated with megakaryocytic differentiation (NF-E2, GATA-1, FOG-1, RUNX-1 and FLI-1) (**d**) are shown. Graphs represent expression levels relative to undifferentiated AND-1_WT. Data represent mean ± standard error of the mean of at least three separate experiments using two AND-1_WT cell lines (AND-1_WT1 and AND-1_WT2) and two AND-1_WASKO (AND-1_WASKO_c1.1 and AND-1_WASKO c1.2). *P < 0.05; **P < 0.01.

We further evaluated the kinetics of emergence of MKs and Plts from AND-1_WT and AND-1_WASKO cells. We found an earlier appearance (**Figure 5a**, top graphs) and increased amounts (**Figure 5a**, bottom graphs; **Supplementary Figure S9**) of MKs and Plts from AND-1_WASKO cells compared to AND-1_WT. Indeed, the percentages and total counts of MKs and Ptls at days 18 and 20 of differentiation were significantly higher for AND-1_WASKO cells but no differences could be detected after day 22. Moreover, the total numbers of MKs and Plts produced by AND-1_WASKO cells during the megakaryocytic culture was two to four times higher to those produced by AND-1_WT cells (**Supplementary Figure S9**). This data is in line with the premature MK differentiation observed by Sabri *et al*. in WASKO mice and indicates that this phenomenon can also occur in humans.

In order to evaluate whether this phenomenon was in part due to an early appearance of MK progenitors, we analyzed the presence of CD34^+^CD41^+^ cells in the EBs at the moment of being plated onto OP9 cells (day 15 of megakaryocytic differentiation). We detected increased levels of CD34^+^CD41^+^ cells in WASKO-EBs (**Figure 5b**). To further corroborate this result, we analyzed the expression levels of two megakaryocytic-specific surface markers (CD41^+^ and CD61^+^) and five transcription factors associated with megakaryocytic differentiation (NF-E2, GATA-1, FOG-1, RUNX-1, and FLI-1) confirming the enhanced MK differentiation of WASKO-EBs compared to WT-EBs (**Figure 5c**,**d**; Black bars versus white bars). All these data together indicate that WASp is controlling megakaryocytic differentiation which is in agreement with data showing premature MK differentiation in mice WASKO models and the accumulation of MKs in bone marrow.^14,37^

### MKs and Plts derived from WASKO hESCs cells mimic the defects found in MKs and Plts from WAS patients

**Figure 6:**
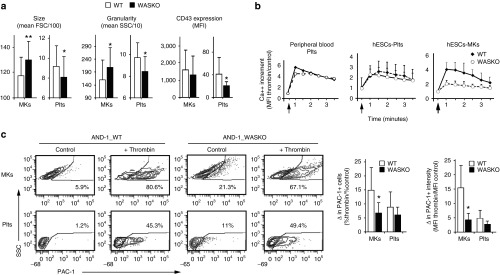
**MKs and Plts from the hESCs-WASKO cell lines mimic functional and phenotypic alterations present in WAS patients**. (**a**) Graphs showing size (FSC; left graphs), granularity (SSC; middle graphs) and CD43 expression (mean fluorescence intensity (MFI); right graphs) of MKs and Plts derived from AND-1_WASKO cells (Black bars) and AND-1_WT (white bars) cells. Data were collected from the CD41^+^CD42^+^ gate. Data are mean ± standard error of the mean of at least six separate experiments using two AND-1_WT cell lines (AND-1_WT1 and AND-1_WT2) and two AND-1_WASKO (AND-1_WASKO_c1.1 and AND-1_WASKO c1.2). *P < 0.05; **P < 0.01. (**b**) Impaired calcium response of AND-1_WASKO MKs. Intracellular calcium levels were evaluated in WASKO (open circles) and WT (black diamonds) Plts obtained from peripheral blood (right graph) or hESCs-cultures (middle graph) as well as in hESC-derived MKs (right braph). WAS patient (L101P on exon 3) peripheral blood was obtained at the Virgen del Rocio Hospital (Sevilla). Healthy donor peripheral blood was obtained as described in Materials and Methods. Ca^++^ flux was measured by flow cytometry using the Calcium Sensor Dye eFluor 514 at a final concentration of 5 μmol/l. Data represent the increase in calcium levels after thrombin stimulation over the basal calcium levels, measured by the intensity of fluorescence of the calcium sensor dye. Thrombin addition (2 U/ml) is indicated by an arrow. (**c**) WASKO-MKs had altered fibrinogen receptor alterations but WASKO-Plts had near-normal responses. AND-1_WT (left plots) and AND-1_WASKO (right plots) were activated with thrombin and stained with CD42 (MK marker) and PAC-1 (antibody that recognizes the dimerized form of this integrin). Plots represent PAC-1 intensity on CD42^+^-gated MKs (top) and Plts (bottom). The graphs show increment in % (left graph) and intensity (right graph) of PAC1^+^ MKs and Pts derived from AND-1_WT (white bars) and AND-1_WASKO (black bars) cells. Data are Mean of at least three separate experiments ± standard error of the mean using two AND-1_WT cell lines (AND-1_WT1 and AND-1_WT2) and two AND-1_WASKO (AND-1_WASKO_c1.1 and AND-1_WASKO c1.2). *P < 0.05; **P < 0.01.

Plts from WAS patients are smaller and have some phenotypic alterations like reduced levels of CD43 at their surface.^15,16,17,18^ However, it is not known whether these abnormalities are already present at the time of Plts emergence from the MKs or if these alterations are acquired in the blood stream. We therefore compared the phenotype of MK and Plts derived from WASKO hESCs with MK and Plts derived from control hESCs. FACS analysis showed that WASKO-Plts were generally smaller (lower FSC) and less granular (lower SSC) than WT-Plts (**Figure 6a**; left and middle graphs-Plts). These data is in agreement with the smaller Plts size found in Plts from WAS patients and in Plts derived from WAS paitents' iPSCs^34^ On the contrary, WASKO-MKs appeared bigger and more irregular than WT-MKs (**Figure 6a**; left and middle graphs-MKs), although this data can reflect a more mature phenotype of WASKO MKs due to the early appearance during the differentiation process. Plts from WAS patients have also reduced CD43 expression levels.^18,38,39^ As observed in WAS patients, WASKO-Plts derived from WASKO hESCs had lower CD43 expression (**Figure 6a**; right graphs-Plts). However, no significant differences were detected in WASKO-MKs.

To study whether WASKO-MKs and WASKO-Plts had altered functionality, we first studied their intracellular Ca^++^ flux (an important regulator of Plts function) after agonist stimulation. As controls, we used Plts from a healthy individual and from a WAS patient (L101P on exon 3) (**Figure 6b**; left graph). Our data showed that Plts derived from hESCs WASKO had near normal intracellular Ca^++^ mobilization after thrombin signaling compared to Plts derived from hESCs WT (**Figure 6b**; middle graph). Similar results were obtained when comparing Plts from peripheral blood of the WAS patient with Plts from a healthy individual (**Figure 6b**; left graph). Interestingly, a tendency to prolong the agonist-induced Ca^++^ flux in WASKO-Plts compared to WT-Plts can be observed both in Plts derived from hESCS WASKO and in Plts from the WAS patient (**Figure 6b**; left and middle graphs, open circles). These observations are in agreement with previous data showing a normal Ca^++^ flux increment and prolongation of the agonist-induced Ca^++^ flux in WAS patients.^19^ However, contrary to WASKO-Plts, WASKO-MKs responded poorly to thrombin (**Figure 6b**; right graph), indicating that WASp could play an important role in MKs function.

We next measured activation of the fibrinogen receptor (measured as PAC-1 binding) of WASKO-MKs and WASKO-Plts compared to WT-MKs and WT-Plts. Our data indicated that the fibrinogen receptor of WASKO-Plts was efficiently activated by thrombin (**Figure 6c**, lower plots and graphs) while WASKO-MKs had this response slightly compromised compared to WT-MKs (**Figure 6c**, upper plots and graphs). Interestingly, both WASKO-Plts and WASKO-MKs had increased PAC-1 binding in basal (unstimulated) conditions compared to WT-Plts and WT-MKs (**Figure 6c** and **Supplementary Figure S10**). These data is in agreement with Gross *et al*.^40^ showing a slight increase rate of aggregation and dense granules secretion in response to agonist of platelets from WAS patients. All these data together indicate a deregulation of the mechanisms of activation of the fibrinogen receptor in WASKO MKs and Plts.

### Ectopic expression of *WAS* in AND-1_WASKO cells restored typical MKs and Plts development and normal MKs function

**Figure 7:**
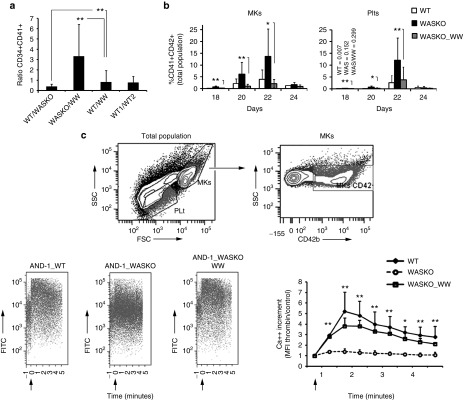
**Ectopic expression of *WAS* on AND-1_WASKO cells restored normal MKs and Plts development and function**. (**a**) Ectopic expression of WAS restores normal levels of CD34+CD41+ progenitors from hESCs-WASKO. The percentages of CD34+CD41+ were determined at day 15 of Megakaryocytic differentiation. The graph shows the ratio between the percentages of CD34+CD41+ cells derived from different hESCs as indicated at the bottom of each bar. Note that a ratio of 1 indicates no differences as observed in the WT/WW (ratio between WT and AND-1_WASKO_WW) and WT1/WT2 (ratio between WT1 and WT2) bars. Data are mean from at least five different experiments ± standard error of the mean. **P < 0.01. (**b**) Ectopic expression of WAS restores normal levels of MKs and Plts generated from hESCs-WASKO. Supernatant from OP9 cocultures from AND-1_WT, AND-1_WASKO, and AND-1_WASKO_WW were collected every 2 days from day 18 and the percentage of MKs (left graph) and Plts (right graph) determined as described in Figure 4. (**c**) Ectopic expression of WAS restores normal calcium responses to AND-1_WASKO_WW derived MKs. Calcium responses to thrombin (arrow) were evaluated in MKs derived from AND-1_WT, AND-1_WASKO, and AND-1_WASKO_WW. The upper plots shows the population considered as MKs. An example of the data generated for each cell line is shown in the bottom-left plots. A graph showing WASKO (open circles), WT (black diamonds) and WASKO_WW MKs (open squares) Ca++ responses along time is shown at the bottom-right corner. Data represent the increase in calcium levels after thrombin stimulation over the basal calcium levels, measured by the intensity of fluorescence of the calcium sensor dye. Thrombin addition (2 U/ml) is indicated by an arrow. Data represent mean ± SEM of at least three separate experiments using two AND-1_WT cell lines (AND-1_WT1 and AND-1_WT2), two AND-1_WASKO (AND-1_WASKO_c1.1 and AND-1_WASKO c1.2) and two AND-1_WASKO_WW (AND-1_WASKO_WW.1 and AND-1_WASKO_WW.2. *P < 0.05; **P < 0.01.

We finally investigated whether ectopic expression of *WAS* in AND-1_WASKO cells could restored the altered phenotypes observed. We generated two bulk populations (AND-1_WASKO_WW.1 and AND-1_WASKO_WW.2) by transduction with the WW-puro vector (see Materials and Methods) and puromycin selection. In both cell lines, we observed low levels of *WAS* expression in undifferentiated hESCs and above normal levels of WAS expression upon hematopoietic differentiation (**Supplementary Figure S11**). We then studied whether the ectopic expression of *WAS* in hESCs-WASKO cells could restored normal megakaryocytic differentiation, as this is one of the most clear but at the same time unexpected phenotype. We therefore performed megakaryocytic differentiation of the AND-1_ WT, AND-1_WASKO and AND-1_WASKO_WW cell lines and studied emergence of CD34+CD41+ progenitors, MKs and Plts. Our data showed that the percentage of CD34+CD41+ cells derived from AND-1_WASKO_WW at day 15 was similar to those derived from AND-1_WT (ration WT/WW = 1; **Figure 7a**) but much lower than those obtained from AND-1_WASKO cells (ratio WASKO/WW = 3 in **Figure 7a**). In the same direction, the AND-1_WASKO_WW cell lines also produced similar levels of MKs (**Figure 7b**, left) and Plts (**Figure 7b**, right) compared to AND-1_WT (white versus gray bars) and lower levels compared to hESCs-WASKO (gray bars versus black bars). In summary, our data showed that the ectopic expression of WAS in AND-1_WASKO cell lines restored normal megakaryocytic development.

We next studied whether the expression of WAS in AND-1_WASKO could also restore normal MKs calcium responses to thrombin. Calcium responses were therefore evaluated in MKs derived from AND-1_WT, AND-1_WASKO and AND-1_WASKO_WW. Our data clearly indicates that the ectopic expression of WAS in AND-1_WASKO restored the ability of MKs to respond to thrombin (**Figure 7c**, lower-left plots and graph; AND-1_WASKO versus AND-1_WASKO_WW; *P* < 0.01).

## Discussion

The present work aims to unveil the defects associated with the absence of WASp during hematopoietic and megakaryocytic development as well as Plts production. A matter still not well understood due to the obvious difficulties in isolation of human hematopoietic progenitors from WAS patients, together with incapability of the WASKO mice in resembling human thrombocytopenia. In addition, these mice models have not been able to detect the genotoxicity of retroviral insertions when used to test gene therapy safety in preclinical studies. Therefore, we were also interested in the development of human cellular models that could be used to study efficiency and safety of new gene therapy strategies for the treatment of Wiskott-Aldrich syndrome. We therefore proposed the development of a human stem cellular model for WAS. Based on the AND-1 cell line^31^ and using ZFNs targeted to the WAS locus,^41^ we have developed two pluripotent cell line clones (AND-1_WASKO_c1.1 and AND-1_WASKO_c1.2) with a complete absence of WASp to mimic WAS patients with a severe phenotype.

WASp expression starts at the first stages of hematopoietic differentiation^1,32^ but little is known about its role in early hematopoiesis. We investigated if *WAS*-deficient hESCs displayed any alterations during early hematopoietic development. WAS patients and animal models exhibit some abnormal hematopoietic parameters.^12,23^ These abnormalities could be explained by defects in proliferation, migration, and/or apoptosis of the different mature hematopoietic lineages rather than due to a defective hematopoietic development. In fact, most of the data generated from WASKO mice indicate that WASp does not play a major role during hematopoietic development. However, recent publications showed increased number of hematopoietic progenitors in the peripheral blood and spleen of WASKO mice.^12,23^ Charrier *et al*.^23^ showed that the total number of progenitors in WASKO mice (taken together CFC from peripheral blood, spleen, and bone marrow) was almost double the total number of progenitors found in WT mice. This is in agreement with our data indicating that the absence of WASp can promote hematopoietic differentiation and generate higher numbers of progenitors and blood cells. A recent publication by Ingrungruanglert *et al*.^34^ reported the development of the first induced pluripotent Stem Cells (iPSCs) from two WAS patients. In this work, the authors did not found alterations in hematopoiesis of the WAS-iPSCs. However, although their data did not reach significance, a similar tendency can be observed in their data (CD34^+^CD45^+^ Average of 0.56% WT-iPSCs versus 0.9% WAS-iPSCs in their **Figure 1d** (ref. 34)) corroborating our results with hESCs WASKO.

The role of WASp in Plts development remains also largely unknown with contradictory results.^12,13^ Different studies showed either, abnormal^14,36^ or normal^15,16,17,20^ megakaryocytic development. Interestingly, Sabri *et al*.^14^ published evidences of premature MK differentiation in WASKO mice. Our data corroborate the idea of an enhanced differentiation toward the megakaryocytic lineage in the absence of WASp favoring the hypothesis that WASp is negatively regulating megakaryocytic differentiation in the absence of the appropriate signals. In agreement with our data, Ingrungruanglert *et al*.^34^ reported enhanced megakaryocytic differentiation in one of their WAS iPSCs (WASX503R-R#1), although this result was not repeated in the other WAS iPSCs they generated.

It is a matter of active discussion whether the microthrombocytopenia observed in WAS patients is due to defective production of Plts in bone marrow or due to Plts depletion by the spleen and other organs. AND-1_WASKO cells produced higher levels of MKs and Plts compared to WT MKs. Importantly, these data was confirmed by demonstrating that the ectopic expression of WAS in AND-1_WASKO cell lines restored normal megakaryocytic development and Plt production. Contrary to our results, Ingrungruanglert *et al*. reported similar (WASQ19X cell lines) or reduced (WASX503R cell lines) Plts production compared to control iPSCs. These contradictory results can be explained by several factors: (i) Ingrungruanglert *et al*. data was obtained at day 24 and at this day of production, we had similar data (**Figure 5a**, day 24). (ii) Ingrungruanglert *et al*. used iPSCs derived from two patients harboring two different mutations, Q19X, which introduce a premature stop codon and X503R, which produce a larger, instable WASp protein. These mutations can render different phenotypes. In addition, the reprogramming factors can induce alterations in the differentiation process. Our two WASKO hESCs lines harbor the same mutation generated by gene edition and can be compared with the original hESCs used to generate the mutations (AND-1).

We finally investigated whether WASKO-HPCs, WASKO-MKs, and WASKO-Plts mimicked the functional defects found previously in HPCs, MK, and Plts from WAS patients and WASKO mice models. Some authors reported reduced clonogenic potential of HPCs from WASKO mice and WAS patients^33^ while others found normal CFU formation capabilities and maintenance of WAS HPCs.^34,42^ We found a slight, although not significant, reduction in the clonogenic potential of WASKO-HPCs compared to WT-HPCs, indicating that the absence of WASp does not play a relevant role in this property of HPCs, at least under this experimental conditions. Mani *et al*.^22^ showed that WASp is involved in kit (SCF-receptor)-mediated signal transduction and required for kit-induced cellular Ca^++^ signaling and survival. Interestingly, we found defective Ca^++^ responses to SCF in WASKO-HPCs compared to WT-HPCs. This finding corroborates Mani *et al*.^22^ data and back up a role of WASp in HPCs survival/maintenance through its function as an effector of SCF signaling.

The phenotypic and functional alterations of MK and Plts from WAS patients are also an open debate. Some authors maintain that Plts function normally but have phenotypic alterations (enhanced phosphatidylserine expression, low CD43 expression) that give rise to its clearance by the spleen.^15,16,17,18^ However, other groups have reported increased basal intracellular Ca^++^ content and prolongation of agonist-induced Ca^++^ flux upon stimulation^19^ as well as reduced activation of the fibrinogen receptor,^20,21^ both important players in Plts function. WASKO-Plts generated from WASKO-hESCs presented the typical phenotypic alterations found in Plts from WAS patients (reduced size and granularity as well as reduced expression levels of CD43). In addition, we confirmed the ability of WASp-deficient Plts to respond to different agonist presenting near-normal Ca^++^ flux and fibrinogen receptor activation. These data are in agreement with the hypothesis that Plts from WAS patients have a near-normal activity and that the recurrent bleeding observed in these patients are due to the thrombocytopenia caused by Plts clearance by resident macrophages in spleen and other tissues. This hypothesis is further confirmed by the restoration of Plts count and coagulation rates in most splenectomized WAS patients.

Interestingly, contrary to the near-normal function observed in WASKO-Plts, WASKO-MKs had significant alterations in both, intracellular Ca^++^ flux and fibrinogen receptor activation upon stimulation with thrombin. Mks require a very structured and dynamic organization of the actin cytoskeleton to generate Plts at the correct place and time. We hypothesized that the absence of WASp deregulate actin cytoskeleton dynamics that became now governed by WASp-homologs (N-WASp, WAWE, etc.) leading to enhanced megakariopoiesis and altered Plts phenotypes.

In summary, we have developed and validated the first human embryonic stem cell model that mimics WAS patients with a severe phenotype. Using these cells, we have demonstrated that WASp is regulating early hematopoietic development, megakaryocytic differentiation, and thrombopoiesis. We have also demonstrated that WASp plays an important role in HPCs and MKs function by showing defective Ca^++^ responses in its absence. Finally, we have demonstrated that these cells can be used to investigate efficiency and safety of gene therapy strategies by restoring WAS phenotype by lentiviral transduction of hESCs WASKO.

## Materials and Methods

***Embryonic stem cell lines culture.*** Human embryonic stem cells AND-1_WT1 (ref. 31) (AND-1; International Stem Cell Registry: Spanish National Stem Cell Bank, Granada, Spain) were cultured in Matrigel (BD Biosciences)-coated T25 flasks in human mesenchymal stem cells-conditioned medium supplemented with 8 ng/ml basic fibroblast growth factor (bFGF) obtained from the Biobanco del Sistema Sanitario Público de Andalucia or onto irradiated human mesenchymal stem cells (Biobanco del Sistema Sanitario Público de Andalucia) in DM medium (KO-DMEM (Invitrogen, Carlsbad, CA), 20% fetal bovine serum (FBS), 1% Glutamax, 1% non-essential aminoacids, and 0.2% β-mercaptoethanol) as previously described.^43^ Approval from the Spanish National Embryo Ethical Committee was obtained to work with hESCs. AND-1_WT2 was generated by cloning the original AND-1_WT1 cells following a similar procedure as for the generation of the WASKO clones but without antibiotic selection.

***Gene edition of hESCs.*** Plasmids encoding the ZFN pairs targeting the *WAS* locus and the plasmid containing the Donor DNA (harboring a Neomycin-resistance expression cassette) were previously published.^41^ The hESCs were pretreated with 10 µmol/l ROCK inhibitor (Millipore, Billerica, MA) for 1 hour, detached with 1 ml of TrypLE (Invitrogen), neutralized with DM medium (KO-DMEM (Invitrogen), 20% FBS, 1% Glutamax, 1% non-essential aminoacids, and 0.2% β-mercaptoethanol)) and resuspended in 100 µl of Amaxa nucleofector solution II (Lonza, Basel, Switzerland). The three plasmids (two ZFNs and one donor DNA) were added to this solution and applied the program A23. The cells were resuspended in 500 µl of prewarmed RPMI medium and recovered for 15 minutes at 37 °C and transferred onto a matrigel-coated 10-cm dish with DM medium containing ROCK inhibitor for 3–4 days. The medium was complemented with 50 µg/ml of G418 for 15 days, refreshing the medium each 2–3 days. Visual screening under the microscope was performed to identify gene-edited colonies. After growth, G418-resistant AND-1 colonies were collected and expanded. The different clones were stored in liquid nitrogen and analyzed for genomic edition by PCR using the NeoF1/WASR1 and the WASF1/WASR1 primer combinations (see **Supplementary Table S1**). Two clones were selected for characterization and analysis (AND-1_ WASKO c1.1 and AND-1_ WASKO c1.1; International Stem cell Registry, Spanish National Stem Cell Bank).

***DNA preparation, RNA preparation, and quantitative real-time PCR.*** Genomic DNA was isolated at the GENYO general service facilities using NucleosSpin Tissue kit (Macherey-Nagel, Düren, Germany). Total RNA was isolated using TRIzol (Invitrogen) following the manufacturer's instructions and reverse transcribed into cDNA using the MultiScribe™ Reverse Transcriptase kit (Applied Biosystems, Foster City, CA). Quantitative real-time PCRs were performed in the Mx3005P Stratagene thermal cycler (Agilent Technologies, Santa Clara, CA) using the QuantiTect SYBR Green PCR Kit (Qiagen, Hilden, Germany). The primers used to quantify relative levels of the different genes are showed in **Supplementary Table S1**.

***Vector production and transduction of hESCs-WASPKO cells.*** The human immunodeficiency virus (HIV) packaging (pCMVΔR8.91) and VSV-G (pMD2.G) plasmids (http://www.addgene.org/Didier_Trono) are described elsewhere.^44^ The lentiviral vector expressing WASP (WW-puro) was obtained by inserting the pSV40-puro cassette from the pPur Vector plasmid into the KpnI site of the WW plasmid^45^ using standard cloning techniques. Vector production and concentration was performed as previously described.^46^ For transduction, hESCs-WASKO cells were incubated with WW-puro lentiviral particles at multiplicity of infection = 10–20. Transduced hESCs-WAKO were incubated under 0.25 μg/ml of puromycine to generate hESCs expressing *WAS*. Two bulk populations (AND-1_WASKO_WW.1 and AND-1_WASKO_WW.2) resistant to puromycine where generated in two different experiments.

***Western blot.*** Cells were lysed with 0.2% NP-40 lysis buffer containing protease inhibitor cocktail (Sigma, St Louis, MO), resolved by sodium dodecyl sulfate (SDS)-polyacrylamide gel electrophoresis (PAGE), electrotransferred to nitrocellulose membranes (Amersham, UK) and probed with anti-human WASp mAb D1 (Santa Cruz Biotechnology, Santa Cruz, CA). For detection, we used goat anti-mouse antibody conjugated with the 800CW infrared dye (1:10,000 dilution) (Li-cor Biotechnologies, Lincoln, NE). The blot was developed by the infrared detection system Odyssey (Li-cor Biotechnologies). Loading controls were carried out by rehybridization of stripped membranes with a rabbit anti-human Erk polyclonal antibody (anti-MAP kinase 1/2, Upstate Biotechnology, UK), followed by incubation with goat anti-rabbit antibody conjugated with the infrared dye 680LT at (1:20,000) (Li-cor Biotechnologies).

***hESCs hematopoietic differentiation through EB.*** Near confluent hESCs (day 0) were treated with collagenase IV for 1 minute, scraped off, transferred to low-attachment plates (Corning Life Sciences, Amsterdam, The Netherland) and incubated overnight in DM medium. The next day, the EBs were centrifuged and resuspended in DM medium supplemented with cytokine cocktail 1 (CC1): BMP-4 (25 ng/ml), Flt-3L (300 ng/ml), SCF (300 ng/ml), IL-3 (10 ng/ml), IL-6 (10 ng/ml), and G-CSF (50 ng/ml) with medium changes every 4 days. EBs were dissociated using collagenase B (Roche Diagnostic, Basel, Switzerland) with Cell Dissociation Buffer (Gibco-Invitrogen) for FACS analysis and CFUs assays. To evaluate hematopoietic differentiation, dissociated cells were stained with 7-AAD (Life Technologies), anti-human CD31-PE, anti-human CD34-PE-Cy7, and anti-human CD45-APC (all from eBiosciences, San Diego, CA). The samples were analyzed in a FACS Canto II flow cytometer equipped with the FACS Diva analysis software (Becton Dickinson, Franklin Lakes, NJ).

***hESCs hematopoietic differentiation in OP9 coculture system.*** Hematopoietic differentiation was induced by transferring the hESCs lines onto OP9 feeders as previously described for at least 15 days (see **Supplementary Figure S4**). To evaluate hematopoietic differentiation at the different days, cells were treated with collagenase IV 1 hour, 10 minutes with Tryple (Gibco), resuspended in phosphate-buffered saline (PBS) 1× +3% FBS + 2 mmol/l ethylenediaminetetraacetic acid buffer, filtered through a 70-µm cell strainer (BD Biosciences, Bedford, MA) and stained with anti-mouse CD29-FITC (AbD Serotec, Raleigh, GBK), anti-human CD31-PE, anti-human CD34-PE-Cy7, and anti-human CD45-APC (all from eBiosciences) and analyzed in a FACS Canto II flow cytometer.

***CFU assay.*** For CFU assays, 20,000–35,000 cells were filtered through a 40-μm cell strainer (BD Biosciences) and directly plated into methylcellulose H4034 (Stem Cell Technologies, Vancouver, Canada). Cells were incubated at 37 °C, 5% CO_2_ humidified atmosphere. The colonies were count based on morphological characteristics after 10–14 days.

***Megakaryocytic differentiation protocol.*** We used a protocol adapted from Lu *et al*.^30^ and is depicted in **Supplementary Figure S5**. The hESCs contained in a T-25 flask were treated with collagenase IV, scrapped, and incubated in DM medium on ultralow attachment plates in order to form EBs (day 0). Twenty-four hours later, the DM medium was complemented with the cytokine cocktail 1 (cc1: BMP4 (50 ng/ml), vascular endothelial growth factor (50 ng/ml), and bFGF (20 ng/ml)) for 2 days (till day 3). From day 3 till day 8, the cytokine cocktail 2 (cc2: SCF, thrombopoietin (TPO), and FLT3 ligand (20 ng/ml each)) were added to the previous cytokine cocktail. Finally, from day 8 to day 15, the EB medium is complemented only with cytokine cocktail 3 (cc3: 350 ng/ml of TPO and 20 ng/ml of SCF) (all cytokines were from PeproTech-London, UK). At day 10 of the EB differentiation protocol, 3 × 10^4^ OP9 cells/well were plated on 0.1% gelatine-coated 6-well plates to form the stroma. On day 15, the EBs were dissociated with 1 ml of collagenase B (Roche, Switzerland) diluted 1:10 in EB medium and collected at 1100 rpm for 2 min. The samples were then incubated with 1 ml of dissociation buffer (Gibco) without pipetting, incubated at 37 °C for 10 minutes, washed with PBS, centrifuged at 1,200 rpm for 4 minutes and resuspended in 1 ml of OP9 differentiation medium (α minimum essential medium basal medium, 10% non-heat-inactivated FBS, 100 μmol/l monothioglycerol, and 50 μg/ml ascorbic acid) containing TPO (100 ng/ml), SCF (50 ng/ml), and heparin (25 U/ml) very carefully until no clumps were seen. Cells were then added onto the OP9 stroma and maintained in this medium during the rest of the differentiation protocol (for another 10–15 days).

To evaluate megakaryocytic differentiation, day 15 EBs from one well of a six-well plate were dissociated, the megakaryocytic progenitors analyzed in 1/5 of the sample and the rest plated again onto one OP9-coated well of a six-well plate. The progenitors were analyzed by staining with 7AAD, mouse anti human CD34-PECy7 (eBiosciences) and mouse anti human CD41-PE (eBiosciences). With the remaining sample, at the indicated days during the differentiation protocol, half of the medium was harvested (containing emerging MKs and Plts) and replaced by fresh medium. Collected samples were spun down at 900 g for 5 minutes, stained in PBS with 3% FBS and 2 mmol/l of Ethylenediaminetetraacetic acid using 7-AAD, mouse anti human CD41-PE (eBiosciences) (1/100 dilution) and CD42b-APC (Biolegend, San Diego, CA) (1/50 dilution) antibodies. Samples were analyzed using a FACSCanto II flow cytometer.

***Isolation of peripheral blood platelets from healthy donors and WAS patients.*** All healthy donors and a WAS patient (L101P on exon 3) gave written informed consent following the Declaration of Helsinki Protocols and the research was approved by the ethical committee of the HU Virgen del Rocio (Sevilla). Blood extractions were performed at the H. Virgen del Rocío. To isolate the Plts, 5 ml of blood were diluted 1/1 in PBS and centrifuged at 300 g for 20 minutes at RT without break. The supernatant containing the Plts were collected and counted.

***PAC-1 binding assay.*** MKs and Plts were collected from the supernatant by centrifugation at 900 g for 5 minutes, washed with Tyrode's buffer (TB) with calcium and magnesium (150 mmol/l NaCl, 2.9 mmol/l KCl, 12 mmol/l NaHCO_3_, 0.1% glucose, 0.1% BSA, 5 mmol/l 4-(2-hydroxyethyl)-1-piperazineethanesulfonic acid, 1 mmol/l CaCl_2_, 1 mmol/l MgCl_2_) and resuspended in 100 µl of the same buffer. The cells were activated with thrombin (2 units/ml) during 10' at RT, stained with the anti-CD42b-APC and PAC-1-FITC antibodies and incubated for 25' at RT. The cell suspension was then washed with TB and fixed in 0.25% PFA in TB.

***Calcium flux.*** For hESCs-derived CD34^+^CD45^+^-derived cells: On day 15 (EBs) or Day 8 (OP9) of differentiation, suspension cells were collected by centrifugation (400 g for 7 minutes) and stained with the anti-CD34-APC antibody for 30 minutes at RT, and washed with TB. Cells were diluted in TB containing the calcium sensor dye eFluor 514 (eBiosciences) at a final concentration of 5 µmol/l. For loading the cells with the dye and establish the background, they were incubated for 40 minutes at 37 °C, then washed and resuspended in TB without dye. Cells were then analyzed by flow cytometry with continuous acquisition for 30 seconds to measure background levels (*T* = 0). Acquisition was then paused to add the SCF (100 ng/ml) and immediately restarted for the continuous acquisition for 6 minutes.

For MKs and Plts: On selected days of differentiation, suspension cells were collected by centrifugation (900 g for 7 minutes), stained with the anti-CD42b-APC antibody for 30 minutes at RT and washed with TB. The rest of the procedure is the same as above but using 2 U/ml of thrombin as stimuli.

***Statistical analysis.*** All data are expressed as mean ± standard error of the mean. Statistical comparisons were performed using the Student *t*-test, with the assumption of normal distribution. Statistical significance was defined as a *P* value < 0.05.

**SUPPLEMENTARY MATERIAL** **Figure S1.** Phenotypic characterization of AND-1_WASKO cell lines. **Figure S2.** AND-1_WASKO_C1.1 generates normal teratomas in an immunodeficient mice model. **Figure S3.** Karyotype of hESCs used. **Figure S4.** hESCs hematopoietic differentiation in OP9 co-culture system. **Figure S5.** Scheme of megakaryocytic differentiation of hESCs. **Figure S6.** MKs and Plts derived from hESCs. **Figure S7.** Characterization of MKs and Plts derived from hESCs. **Figure S8.** Characterization of Plts from Peripheral blood. **Figure S9.** AND-1_WASKO cells produce higher number of MKs and Plts. **Figure S10.** WASKO-MKs had increased basal levels of PAC-1 binding. **Figure S11.** WW-puro LVs Transduced AND-1_WASKO cell express WAS. **Figure S12.** Plots showing the population analyzed to measure hematopoietic differentiation on the EB system **Table S1.** List of primers used for qPCR and RT-qPCR.
