# Supplementary material for: Absence of WASp Enhances Hematopoietic and Megakaryocytic Differentiation in a Human Embryonic Stem Cell Model
Source: Mol Ther. 2015 Dec 8;24(2):342–53. doi: 10.1038/mt.2015.196 (PMC4817813; doi:10.1038/mt.2015.196)

LEGENDS TO SUPPLEMENTARY FIGURES

Figure S1. ***Phenotypic characterization of AND-1_WASKO cell lines.*** AND-1_WASKO_C1.1 (WASKO_C1.1) and AND-1_WASKO_C1.2 (WASKO_C1.2) were stained with the antibodies oct3/4 (BD Pharmingen), ssea-4 and tra-1-60 (eBiosciences). Live cells were identified by 7-AAD viability dye exclusion. Top panels indicate the double gated population used for analysis of the different markers; FSC/SSC gated population (left) and 7AAD-negative (right). Samples were acquired and analyzed in a FACS Canto II flow cytometer equipped with the FACS Diva analysis software (Becton Dickinson, Franklin Lakes, NJ). Numbers at the right-top corner of each plot represent the percentage of positive cells related to the isotype control.

Figure S2. ***AND-1_WASKO_C1.1 generates normal teratomas in an immunodeficient mice model***. Histological analysis of teratomas developed in NOD/SCID-IL2R$\gamma^{-/-}$ mice 8–10 weeks after inoculation of AND-1_WASKO_C1.1 cells under the testicular capsule. A) Immunostainning of tissue sections for markers of the ectoderm (left panel; β-tubulin), mesoderm (middle panel; smoth muscle actin) and endoderm (right panels; cytokeratin) B) Hematoxylin/eosin staining of teratomas sections showing structures typical of bone, cartilage and epithelium. These studies were performed at Biobanco of Andalucia (http://www.juntadeandalucia.es/salud/biobanco/).

Figure S3. ***Karyotype of hESCs used***. The hESC line used to obtain the WASKO hESCs lines was AND-1_WT1 (30) (AND-1; Spanish Stem Cell Bank. www.isciii.es). AND-1_WT2 was derived by dilution of AND-1_WT1 and collection of individual

colonies without any further manipulation. AND-1_WASKO_C1.1 and AND-1_WASKO_C1.2 were generated as described and M&M. A representative G-banding analysis of 20-30 metaphase spreads per hESCs line was performed at Biobanco of Andalucia (http://www.juntadeandalucia.es/salud/biobanco/). The result of the study showed no additional alterations compared with the original AND-1_WT1 line from passage 10. This hESC line presents an abnormal karyotype with the loss of a chromosome 7 and an isochromosome gain corresponding to the p arm of this chromosome.

Figure S4. ***hESCs hematopoietic differentiation in OP9 co-culture system.*** (**a**) Scheme depicting the protocol for hematopoietic differentiation using OP9 system. Hematopoietic differentiation was induced by transferring the hESCs lines onto OP9 feeders for at least 15 days. To evaluate hematopoietic differentiation at the different days, cells collected from the supernatant were resuspend in PBS1x +3%FBS+ 2mM EDTA buffer, filtered through a 70-µm cell strainer (BD Biosciences, Bedford, MA) and stained with anti-mouse CD29-FITC (AbD Serotec, Raleigh, GBK), anti-human CD34-PE-Cy7 and anti-human CD45-APC (all from eBiosciences, San Diego, CA). Hematopoietic populations were analyzed by Flow Cytometry (FC) at different days of differentiation as indicated. (**b**) Plots showing the population analyzed to measure hematopoietic differentiation on the OP9 system. Cells were gated based on morphology (FSC/SSC; top-left), viability (7-AAD negative; Top-right) and excluding OP9 cells (CD29 negative; bottom-left). An example of CD34 and CD45 staining on this population is showed (bottom-right) (**c**) Analysis of the $CD34^{+}CD45^{+}$ (Hematopoietic precursor cells) of AND-1_WT and AND-1_WASKO cells at different time points during the hematopoietic differentiation process. Data are average of at least

8 separate experiments using two AND-1_WT cell lines (AND-1_WT1 and AND-1_WT2) and two AND-1_WASKO (AND-1_WASKO_c1.1 and AND-1_WASKO c1.2). * $p<0.05$; ** $p< 0.01$. Significance related to same day on AND-1_WT

Figure S5. ***Scheme of megakaryocytic differentiation of hESCs***. This differentiation protocol is based in Lu S. J., et al (34) and is divided in two stages: an EB differentiation stage (from day 0 to 15) in the presence of human cytokines and an OP9 co-culture differentiation stage (from day 15 to 32) were dissociated EB cells are cultured over OP9 stromal cells supplemented with heparin, human TPO and human SCF (see Supplementary M&M for detail).

Figure S6. MKs and Plts derived from hESCs. Day 20 megakaryocytic cultures from AND-1_WT (top) and AND-1_WASKO (bottom) were photographed on the tissue culture plates with a microscope Primo Vert (Carl Zeiss). The photographs show the Plts-like structures released from the MKs. MKs are in suspension in the supernatant of the co-culture (dissociated EBs and OP9 cells). MKs- (green arrows) and Plts (White arrows)-like structures are indicated. Areas containing MK and Plts are magnified for visualization of Plts (Blue arrow).

Figure S7. Characterization of MKs and Plts derived from hESCs. (**a**) AND-1_WT and AND-1_WASKO cells at day 20 of the megakaryocytic cultures were bound to fibrinogen coated slides, activated with thrombin and immune-stained with Dapi (Blue), Phalloidin (red) and CD61 (green). Three (WASKO-MK1 to WASKO-MK3) or four (WT-MK1 to WT-MK4) different MKs are shown. A Merge image is shown at the right-bottom of each set. (**b**) AND-1_WT and AND-1_WASKO cells were treated as

above and immune-stained with Dapi (Blue), Phalloidin (red) and anti-Vinculin (green). Low magnification images of WASKO-MK (top) and WT-MKs (bottom) are shown. A merged image is shown at the top-left corner of each set and a higher magnification image (represented by the white square and white arrow) is shown in the right-bottom corner. (**c**) Plts derived from AND-1_WASKO (left panels) AND-1_WT (right panels) at day 20 of the megakaryocytic cultures were bound to fibrinogen coated slides, activated with thrombin and immune-stained with CD61 (green) and Phalloidin (red). A Merge image is shown at the right of each set. (**d**) Plts derived from AND-1_WASKO (left panels) AND-1_WT (right panels) at day 20 of the megakaryocytic cultures were treated as above and immune-stained with anti-vinculin (green) and Phalloidin (red). A Merge image is shown at the right of each set. Confocal images were capture with a confocal microscope Zeiss LSM 880

Figure S8. Characterization of Plts from Peripheral blood. Ptls obtained from a healthy donor were bound to fibrinogen coated slides, activated with ADP and immune-stained with Phalloidin (red)/vinculin(green). The panels show typical trapezoidal morphology of activated Plts that are positive for vinculin and f-actin.

Figure S9. AND-1_WASKO cells produce higher number of MKs and Plts. The graph shows the adding up of $CD41^{+}CD42^{+}$ MKs and Plts produced by AND-1_WT (White bars) and AND-1_WASKO (Black bars) lines during all the collection days of the differentiation protocol from a 9.5cm2 well (from a 6-well plate). Data represent average ± SD of three separate experiments. * $p<0.05$; ** $p< 0.01$.

Figure S10. WASKO-MKs had increased basal levels of PAC-1 binding. Plots represent PAC-1 mean fluorescence intensity (MFI) on $CD41^{+}$-gated MKs (left) and Plts (right) derived from AND-1_WT (white bars) and AND-1_WASKO (black bars) cells measured before activation. Data are average of at least three separate experiments +/- standard deviation. * $p<0.05$.

Figure S11. WW-puro LVs Transduced AND-1_WASKO cell express *WAS*. AND-1_WASKO cells were transduced with the WW-puro vector and selected with puromycine to generate WASKO_WW-1 and WASKO_WW-2 cell lines. mRNA from undifferentiated (white bars) or day 22 EB-differentiated AND-1_WT, AND-1_WASKO and AND-1_WASKO_WW cells were isolated and analyzed by q-RT-PCR. The graph shows the relative quantity ($2^{-\Delta\Delta Ct}$) of *WAS* mRNA levels using undifferentiated AND-1 cells as calibrator and GAPDH to normalize the different sample.

Figure S12. Plots showing the population analyzed to measure hematopoietic differentiation on the EB system. Cells were gated based on morphology (FSC/SSC; left plot) and viability (7-AAD negative; right plot)

SUPPLEMENTARY TABLE

Table S1

**Primer name**	**Sequence**	**Accession number**	**Location**
**WASF1**	5’-AAGCACTCACGATAGGCGTGG- 3’	NG_007877	4214
**WASR1**	5’-AAGTTCAGGTCAGGGGATTGTGG- 3’	NG_007877	6182
**NeoF1**	5’- TGCTCCTGCCGAGAAAGTAT- 3’	KJ619486.1	2844
**F-WAS**	5´-AGGCTGTGCGGCAGGAGAT- 3’	NM_000377	E9
**R-WAS**	5’-CAGTGGACCAGAACGACCCTTG- 3’	NM_000377	E10
**F-CD45**	5’-CCCCCACTGGATTGACTACAG-3’	NM_002838.4	E2
**R-CD45**	5’-GGTGCTTGCGGGTGAGAAT- 3’	NM_002838.4	E2
**F-CD41a**	5’-TGAGCCGCATTTACGTGGAAA- 3’	NM_000419.3	E4
**R-CD41a**	5’-CTTCACAGTAACGCTTGTCCC- 3’	NM_000419.3	E5
**F-CD61**	5’-GTGACCTGAAGGAGAATCTGC- 3’	NM_000212	E3
**R-CD61**	5’-TCACTCACTGGGAACTCGATG- 3’	NM_000212	E3
**F-NFE2**	5’- GCAGGAACAGGGTGATACAGC- 3’	NM_001136023	E2
**R- NFE2**	5’-GAGCAGGGGCAGTAAGTTGT- 3’	NM_001136023	E3
**F-GATA1**	5’- TCACTCCCTGTCCCCAATAG- 3’	NM_002049	E3
**R-GATA1**	5’- GGAGAGTTCCACGAAGCTT- 3’	NM_002049	E3
**F-SCL**	5’- GGATGCCTTCCCTATGTTCA- 3’	NM_003189.5	E5
**R-SCL**	5’- GGTGTGGGGACCATCAGTAA- 3’	NM_003189.5	E6
**F-CD34**	5’- TGGACCGCGCTTTGCT- 3’	NM_001025109.	E1
**R-CD34**	5’- CCCTGGGTAGGTAACTCTGGG- 3’	NM_001025109.	E2
**R-FOG1**	5’-CTGTCCTTCCAGCTCCTTGG- 3’	NM_153813	E3
**F-RUNX1**	5’- CCGAGAACCTCGAAGACATC- 3’	NC_000021	E5
**R-RUNX1**	5’- GCTGACCCTCATGGCTGT- 3’	NC_000021	E6
**F-FLI1**	5’- CCAACGAGAGGAGAGTCATCG- 3’	NM_001167681	E4
**R-FLI1**	5’- TTCCGTGTTGTAGAGGGTGGT- 3’	NM_001167681	E5
**F-GAPDH**	5’-ATGGGGAAGGTGAAGGTCG- 3’	NM_002046.3	E2
**R-GAPDH**	5’-GGGGTCATTGATGGCAACAATA- 3’	NM_002046.3	E3

Fig. S1

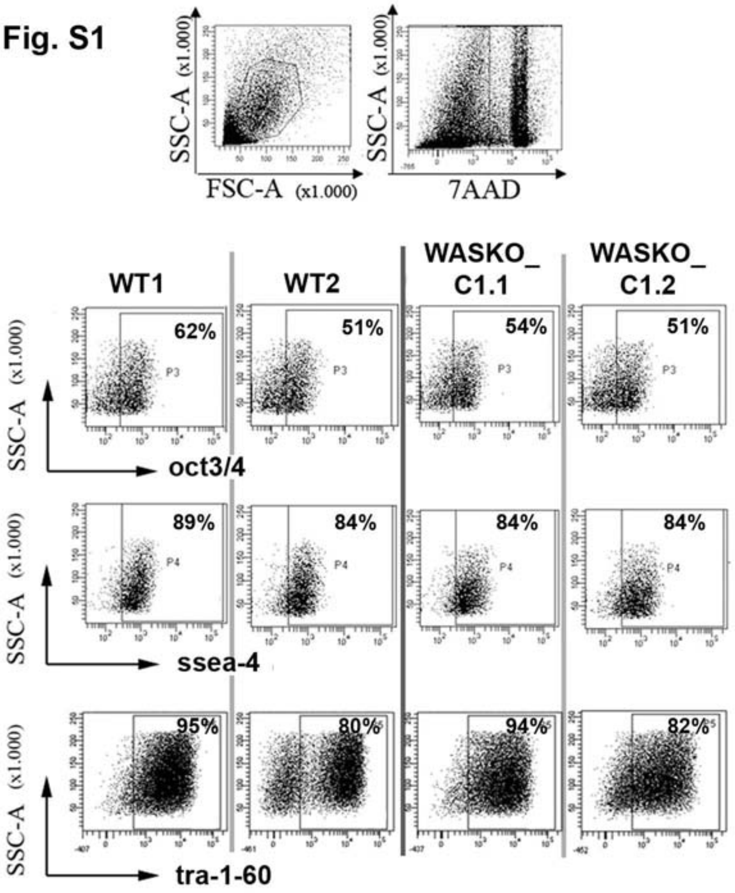

SSC-A (x1.000)
FSC-A (x1.000)
7AAD
WT1
WT2
WASKO_ C1.1
WASKO_ C1.2
62%
51%
54%
51%
P3
oct3/4
89%
84%
84%
84%
P4
ssea-4
95%
80%
94%
82%
tra-1-60

Fig. S2

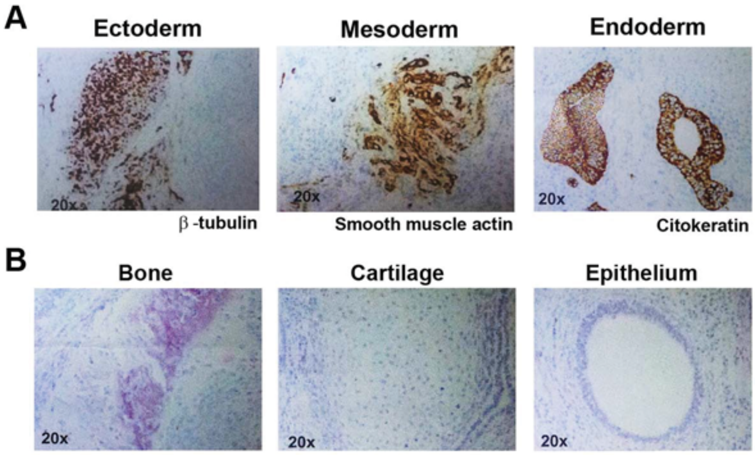
A
Ectoderm
Mesoderm
Endoderm
20x
20x
20x
β -tubulin
Smooth muscle actin
Citokeratin
B
Bone
Cartilage
Epithelium
20x
20x
20x

Fig. S3

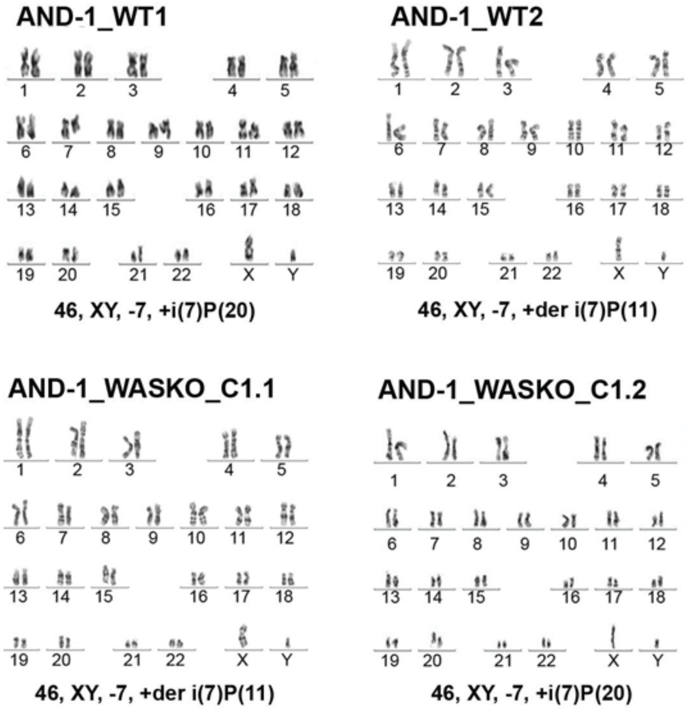
AND-1_WT1
46, XY, -7, +i(7)P(20)
AND-1_WT2
46, XY, -7, +der i(7)P(11)
AND-1_WASKO_C1.1
46, XY, -7, +der i(7)P(11)
AND-1_WASKO_C1.2
46, XY, -7, +i(7)P(20)

Fig. S4

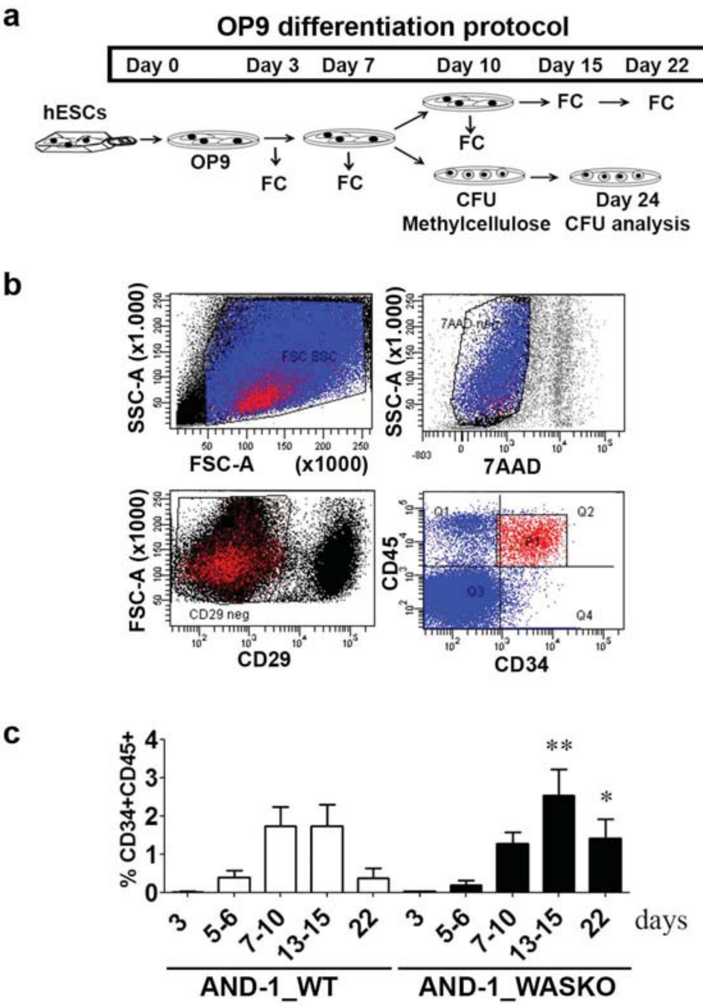
a
OP9 differentiation protocol
Day 0
Day 3
Day 7
Day 10
Day 15
Day 22
hESCs
OP9
FC
CFU
Methylcellulose
Day 24
CFU analysis
b
SSC-A (x1.000)
FSC-A
(x1000)
FSC SSC
7AAD neg
7AAD
FSC-A (x1000)
CD29 neg
CD29
CD45
CD34
Q1
Q2
Q3
Q4
P1
c
% CD34+CD45+
3
5-6
7-10
13-15
22
days
AND-1_WT
AND-1_WASKO
**
*

Fig. S5

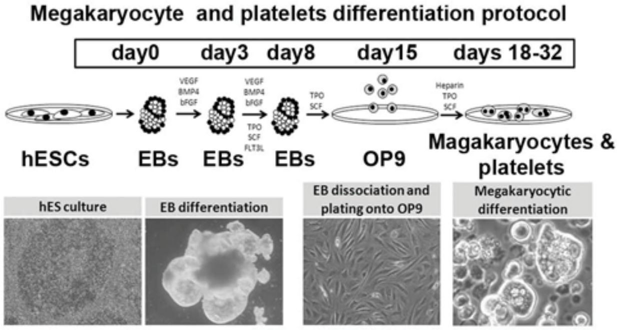
Megakaryocyte and platelets differentiation protocol
day0
day3
day8
day15
days 18-32
VEGF
BMP4
bFGF
VEGF
BMP4
bFGF
TPO
SCF
FLT3L
TPO
SCF
Heparin
TPO
SCF
hESCs
EBs
EBs
EBs
OP9
Magakaryocytes & platelets
hES culture
EB differentiation
EB dissociation and plating onto OP9
Megakaryocytic differentiation

Fig. S6

AND-1_WT1 MKs and PLTs

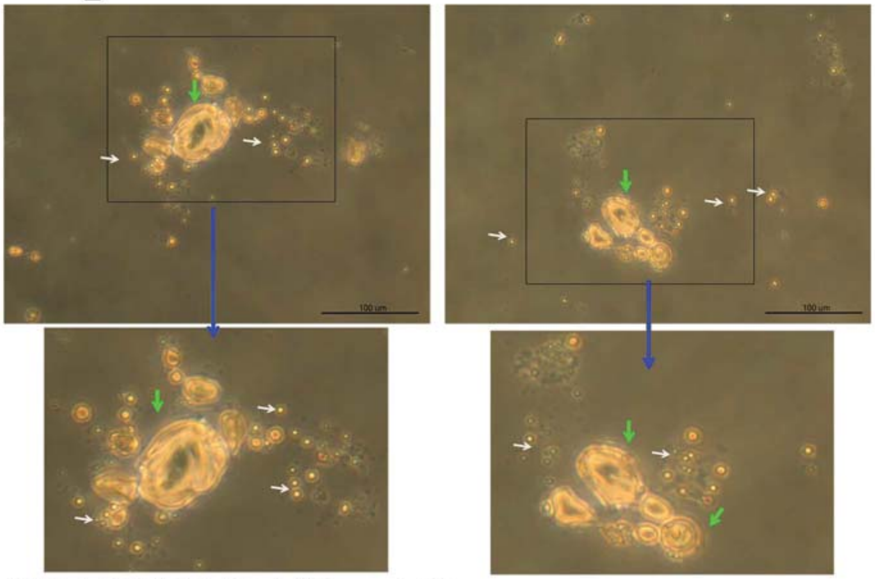

AND-1_WASKO_C1.1 MKs and Plts

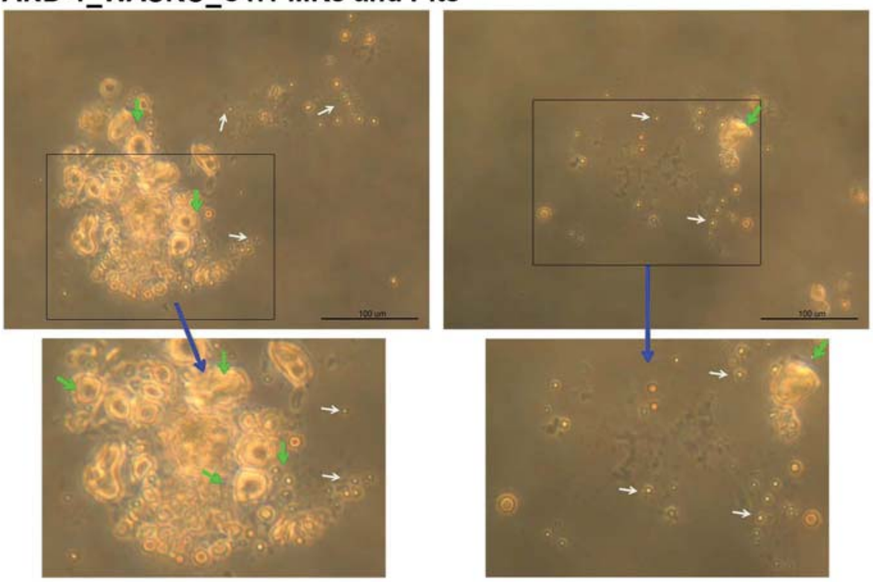

Fig. S7a

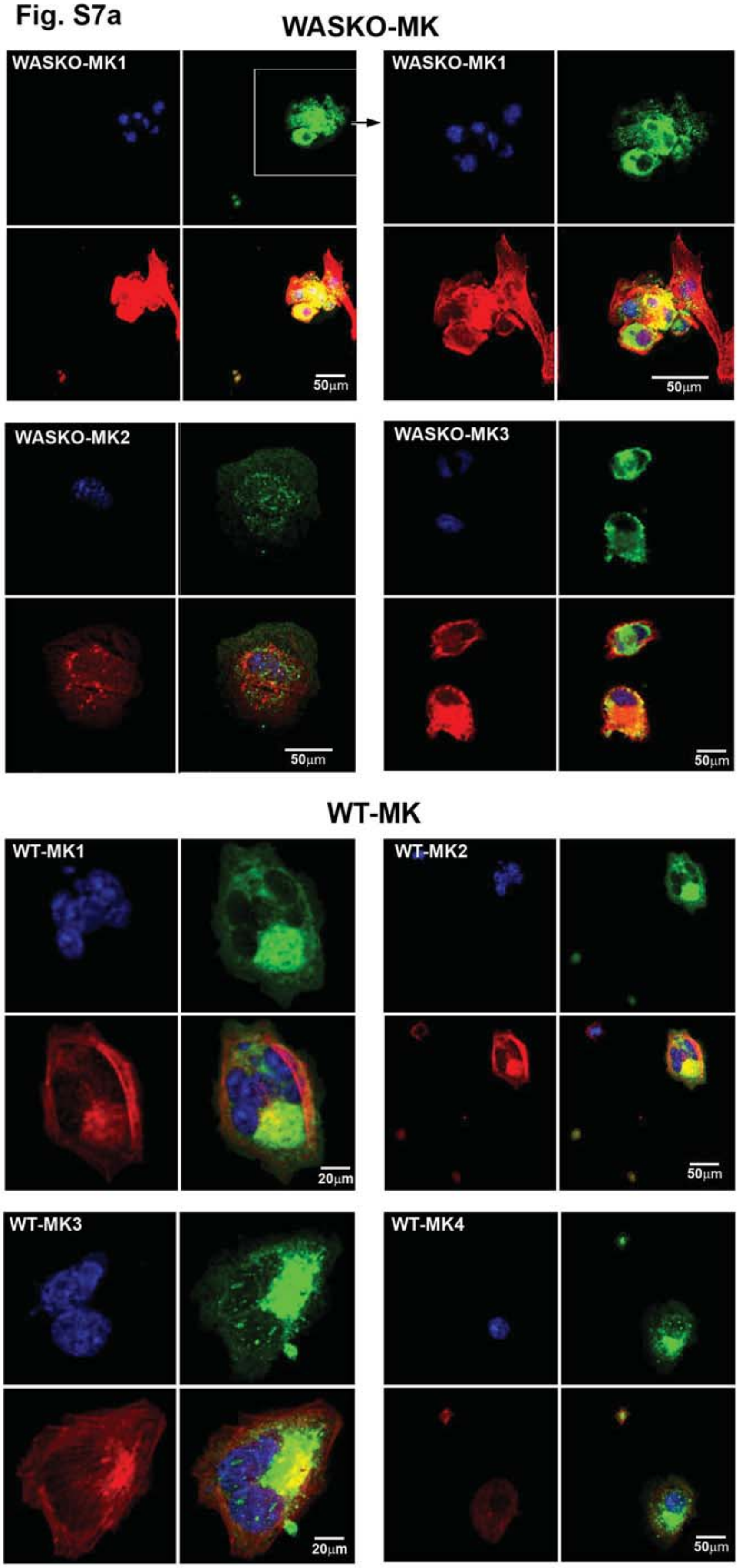

Blue = Dapi Green = α-CD61 Red = phalloidin

Fig. S7b

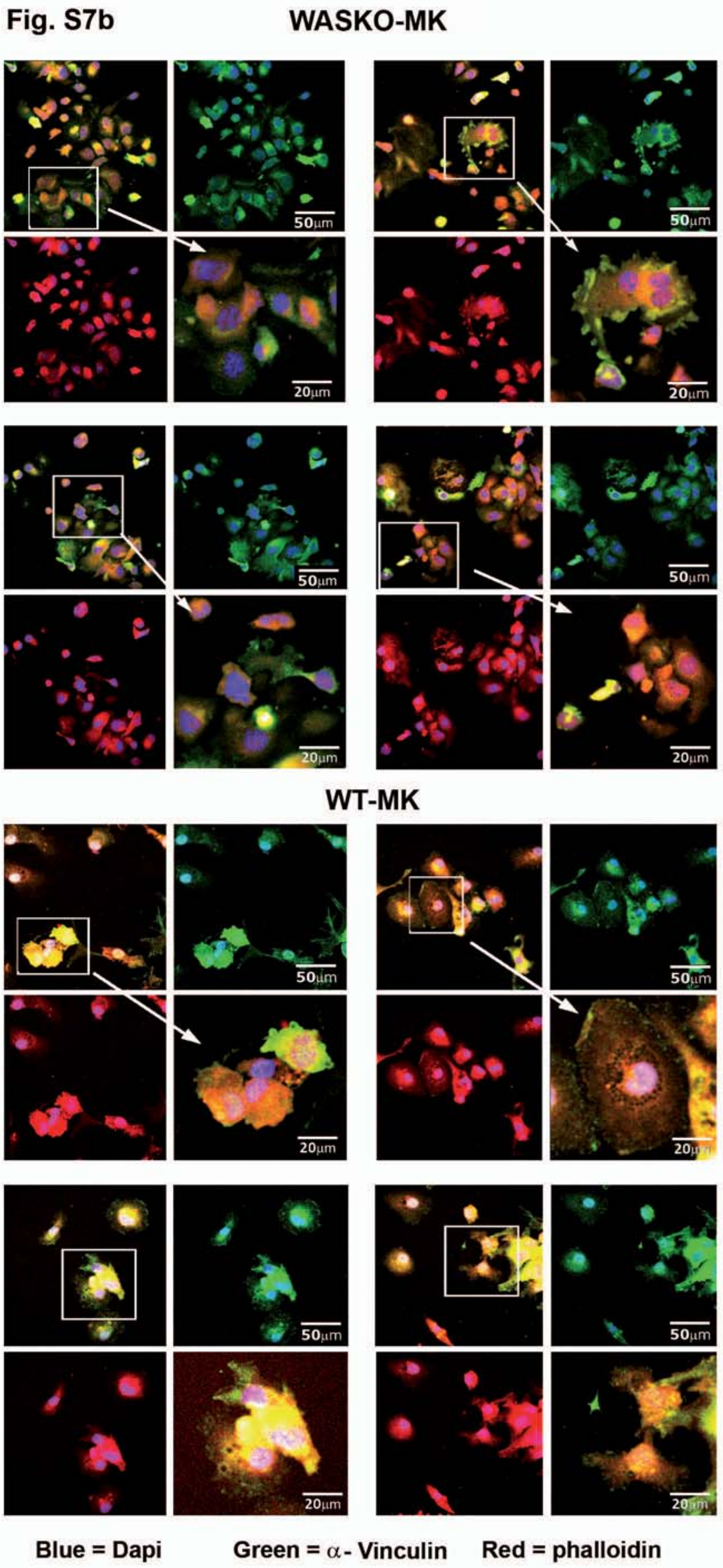
WASKO-MK
50μm
20μm
WT-MK
Blue = Dapi
Green = α- Vinculin
Red = phalloidin

Fig. S7c

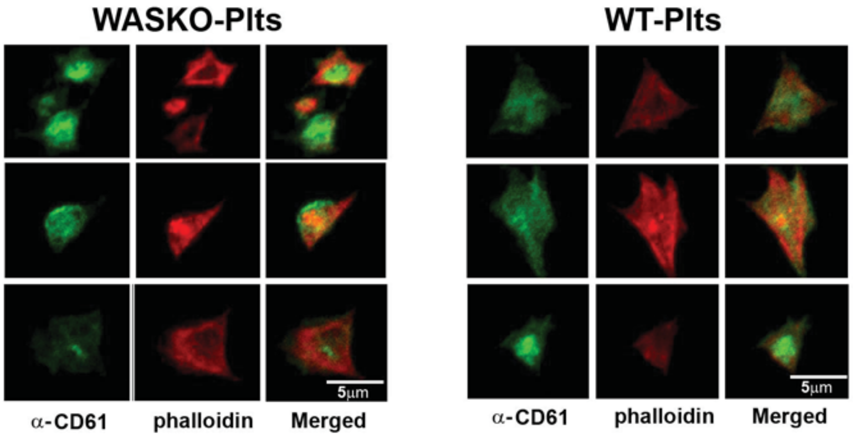
WASKO-Plts
WT-Plts
5μm
5μm
α-CD61
phalloidin
Merged
α-CD61
phalloidin
Merged

Fig. 7d

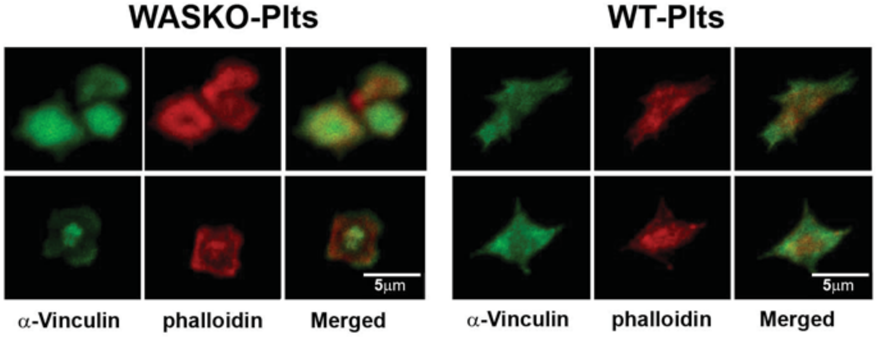
WASKO-Plts
WT-Plts
5μm
5μm
α-Vinculin
phalloidin
Merged
α-Vinculin
phalloidin
Merged

Fig. S8

Peripheral blood Plts

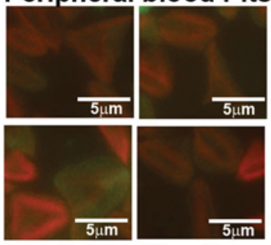

Green = Vinculin
Red = Phalloidin

Fig. S9

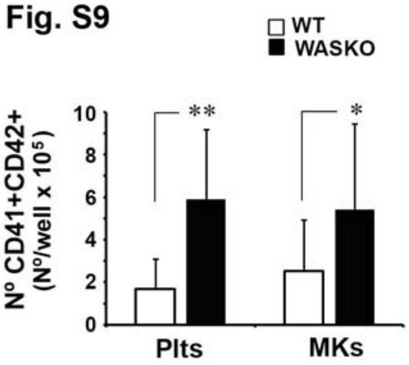

Fig. S10

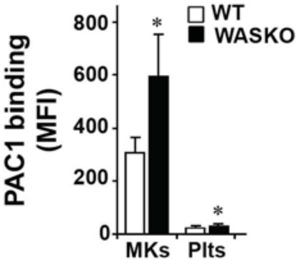

Fig S11

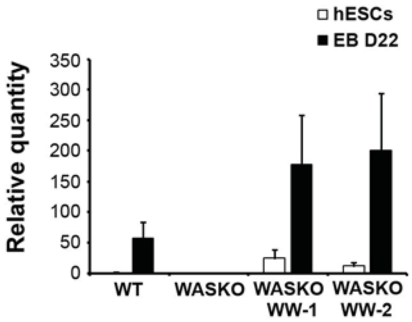

hESCs
EB D22
Relative quantity
350
300
250
200
150
100
50
0
WT
WASKO
WASKO WW-1
WASKO WW-2

Fig S12

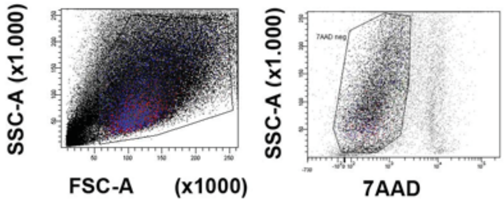

Supplement: Supplementary Figures and Table [file mt2015196x1.pdf]
